# Reconstruction and *in silico* analysis of an *Actinoplanes* sp. SE50/110 genome-scale metabolic model for acarbose production

**DOI:** 10.3389/fmicb.2015.00632

**Published:** 2015-06-25

**Authors:** Yali Wang, Nan Xu, Chao Ye, Liming Liu, Zhongping Shi, Jing Wu

**Affiliations:** ^1^Wuxi Medical School, Jiangnan UniversityWuxi, China; ^2^State Key Laboratory of Food Science and Technology, Jiangnan UniversityWuxi, China; ^3^Carbohydrate Chemistry and Biotechnology, Ministry of Education, Jiangnan UniversityWuxi, China

**Keywords:** *Actinoplanes* sp. SE50/110, genome-scale metabolic model, acarbose production, amino acids, oxygen uptake rate

## Abstract

*Actinoplanes* sp. SE50/110 produces the α-glucosidase inhibitor acarbose, which is used to treat type 2 diabetes mellitus. To obtain a comprehensive understanding of its cellular metabolism, a genome-scale metabolic model of strain SE50/110, *i*YLW1028, was reconstructed on the bases of the genome annotation, biochemical databases, and extensive literature mining. Model *i*YLW1028 comprises 1028 genes, 1128 metabolites, and 1219 reactions. One hundred and twenty-two and eighty one genes were essential for cell growth on acarbose synthesis and sucrose media, respectively, and the acarbose biosynthetic pathway in SE50/110 was expounded completely. Based on model predictions, the addition of arginine and histidine to the media increased acarbose production by 78 and 59%, respectively. Additionally, dissolved oxygen has a great effect on acarbose production based on model predictions. Furthermore, genes to be overexpressed for the overproduction of acarbose were identified, and the deletion of *treY* eliminated the formation of by-product component C. Model *i*YLW1028 is a useful platform for optimizing and systems metabolic engineering for acarbose production in *Actinoplanes* sp. SE50/110.

## Introduction

Type 2 diabetes mellitus is a chronic disease that affects more than 320 million people worldwide, it is the fourth or fifth leading cause of death in most developed countries, and its incidence is rising gradually (Federation, 2003). Additionally, complications from diabetes, such as coronary artery and peripheral vascular disease, stroke, diabetic neuropathy, amputations, renal failure, and blindness reduce people's quality of life (Federation, 2003). There are three kinds of effective drugs for treating type 2 diabetes mellitus: (1) biguanide, which increases the sensitivity to insulin; (2) sulfonylurea, which promotes the secretion of serum insulin, and (3) α-glucosidase inhibitors, such as acarbose and voglibose, which reduce the sugar concentration in the blood. Because of its advantages of mild and persistent therapeutic effects and lack of toxicity, the use of acarbose is increasingly favored for patients, especially for those who consume starchy foods as a staple.

Starting in 1990, the industrial production of acarbose was performed using improved derivatives of the wild-type producer *Actinoplanes* sp. SE50 (ATCC 31042; CBS 961.70). Since then, laborious, conventional mutagenesis and screening experiments were conducted to develop strains with higher acarbose production. Some strains with which acarbose were produced at the bench scale in recent years are listed in Table 1. *Actinoplanes* sp. SE50 was screened from soil by Bayer AG (Frommer et al., 1977), and other strains went through laborious, conventional mutagenesis and screening, followed by fermentation optimization (Choi and Shin, 2003; Wang et al., 2011; Xue et al., 2013; Cheng et al., 2014). Because maltose has been reported to be a moiety of acarbose, which was directly attached to dTDP-acarviose-7-P, and it worked as a carbon source, the carbon sources for *Actinoplanes* sp. CKD485-16, *Actinoplanes* sp. AC-N1, and *Actinoplanes* sp. ZJB-08196 were maltose and glucose. For *Actinoplanes* sp. SE50 and *Actinoplanes* sp. A56, carbon sources were starch and glucose, as maltose could be produced via the hydrolytic activity of the amylases AcbD and AcbE. It is worth noting that in strain *Actinoplanes utahensis* ZJB-08196, which was developed from *Actinoplanes* sp. through ion beam implantation, acarbose production reached 6606 mg/L after optimization of the medium composition and culture conditions (Wang et al., 2011), such as maintaining an elevated osmolality via intermittent feeding of necessary components (14.0 g/L maltose, 6.0 g/L glucose, and 9.0 g/L soybean meal, with feeding at 48, 72, 96, and 120 h and a feed volume of 5 mL) (Wang et al., 2012), as well as the addition of S-adenosylmethionine (12 h post-inoculation, 100 μmol/L S-adenosylmethionine) (Sun et al., 2012) and validamine (prior to inoculation, 20 mg/L) (Xue et al., 2013). It is the highest titer of fermentation by *A. utahensis* until now. Despite this, with the increasing population of diabetics, a higher acarbose yield is urgently required; however, some deficiencies limit improvements of acarbose production. (1) Conventional random mutation and screening, followed by optimization of media composition and culture conditions, often requires tremendous effort, and it has reached its limit. (2) Some metabolic mechanisms involved in the production of acarbose in *Actinoplanes* were unclear; thus, the major bottleneck in acarbose fermentation is still unknown. (3) Very little is known about the overexpression or knockout of gene targets to improve acarbose production at the system level. Therefore, a systematic understanding of physiological features and a global understanding of metabolism are greatly needed. In this regard, genome-scale metabolic model (GSMM) plays an important role in fermentation optimization and metabolic engineering.

**Table 1:** **Strains used to produce acarbose and the acarbose production**.

**Strain**	**Carbon source**	**Production (mg/L)**	**Reference**
*Actinoplanes* sp. SE50	Starch, glucose	1000	Frommer et al., 1977
*Actinoplanes* sp. CKD485-16	Glucose, maltose	3200 (30-L)	Choi and Shin, 2003
*A. utahensis* ZJB-08196	Glucose, maltose	6606 (50-mL)	Wang et al., 2011; Xue et al., 2013
*Actinoplanes* sp. A56	Starch, glucose	4133 (50-L)	Cheng et al., 2014

*Actinoplanes* sp. SE50/110 is known to be the wild-type producer of the α-glucosidase inhibitor acarbose, and because its complete genome sequence is known (Schwientek et al., 2012), it is feasible to construct a GSMM, which enables us to obtain a comprehensive understanding of its global metabolism, thereby aiding the design of metabolic engineering strategies and decreasing the number of wet lab experiments. In this study, the first GSMM of *Actinoplanes*, *i*YLW1028, was constructed, and it was used to elucidate the physiological characteristics of *Actinoplanes* sp. SE50/110 in detail. Strategies to improve acarbose production were simulated based on model *i*YLW1028.

## Materials and methods

### Model reconstruction and refinement

The availability of the whole genome of *Actinoplanes* sp. SE50/110 (Schwientek et al., 2012) enabled us to conduct the model reconstruction following the general process of GSMM reconstruction, which was performed as described previously (Zou et al., 2013). To construct this model, two methods were employed: (1) an auto-reconstructed model was generated from the Model-SEED database; (2) *Bacillus megaterium* WSH002 and a closely related species, *Streptomyces coelicolor*, were compared to *Actinoplanes* sp., and the reaction list was obtained through a local sequence similarity search (BLASTp). The three thresholds in BLASTp were an amino acid sequence identity ≥35%, an *e*-value ≤10^−6^, and a matching length ≥70% of the query sequence. Afterwards, the reaction list from the auto-reconstructed model and BLASTp were combined in Excel with the same format, which formed the draft model. Additionally, some reactions from the literature were supplemented into the draft model, and the biosynthesis pathway of acarbose was included. Public databases, such as KEGG, Metacyc, BiopathExplore, BRENDA, CELLO, TCDB (Saier et al., 2009), and the COBRA software package, were used to refine the draft model, including filling metabolic gaps, balancing mass and charge, determining reaction directionality, adding information for gene and reaction localization, adding subsystem information to reactions, and adding some transport reactions.

### Organism and cultivation conditions

Strain *Actinoplanes* sp. SE50/110 was purchased from the ATCC.

Sucrose medium contained 30 g/L sucrose, 2.0 g/L peptone, 1.0 g/L casein hydrolysate, 1.0 g/L K_2_HPO_4_.3H_2_O, 0.5 g/L KCl, 0.5 g/L MgSO_4_.7H_2_O, and 0.1 g/L FeSO_4_.7H_2_O, 2 g/L agar. The minimal medium for the functional test contained a carbon source (20 g/L), a nitrogen source (5 g/L), 1.0 g/L K_2_HPO_4_.3H_2_O, 0.5 g/L KCl, 0.5 g/L MgSO_4_.7H_2_O, and 0.1 g/L FeSO_4_.7H_2_O, and the initial pH was adjusted to 7.0. The medium used for seed cultures was NBS medium, which contained 11 g/L glucose, 4 g/L peptone, 4 g/L yeast extract, 1 g/L MgSO_4_.7H_2_O, 2 g/L KH_2_PO_4_, and 5.2 g/L K_2_HPO_4_.3H_2_O (Wendler et al., 2013). The acarbose synthesis medium was based on high-maltose minimal medium (Wendler et al., 2013). It consisted of two solutions. Solution 1 consisted of 70 g/L maltose·1H_2_O, 5 g/L (NH_4_)_2_SO_4_, 6.55 g/L K_2_HPO_4_·3H_2_O, 5 g/L KH_2_PO_4_, 5.7 g/L trisodium citrate·2H_2_O, 1.0 g/L MgCl_2_·6H_2_O, and 2.0 g/L CaCl_2_·2H_2_O. Solution 2 contained trace elements, including 15.75 mM FeCl_2_, 25.00 mM MnCl_2_, 33.75 mMCaCl_2_, 3.75 mM ZnCl_2_, 0.50 mM CuCl_2_, 0.05 mM NiCl_2_, and 0.02 mM [Co(NH_3_)_6_]Cl_3_. Solution 1 was sterilized by moist heat, while solution 2 was filter sterilized, and afterwards, 0.2 mL of solution 2 was added to solution 1.

To prepare for the fermentation process, the strain was cultured on a sucrose medium for 72 h. Next, seed cultures were prepared by two sequential seed cultures. The first seed cultures were prepared by transferring a colony of about 1 × 1 cm^2^ to 50 mL NBS medium in a 500 mL Erlenmeyer flask, followed by culturing for 50 h at 28°C with shaking at 200 rpm. A 5 mL aliquot of first seed cultures were used to inoculate 50 mL of second seed medium in a 500 mL Erlenmeyer flask (equivalent to 10% of the inocula), and the seed cultures were formed. Subsequently, the seed cultures were centrifuged for 6 min at 6000 × g, and then washed twice with 0.9% NaCl solution. After another wash and centrifugation step (6 min, 6000 × g), the supernatant was decanted and the resulting pellet was resuspended in 50 mL NaCl solution, of which 5 mL (equivalent to 8% of the inocula) was inoculated into 50 mL of acarbose synthesis medium in 500 mL flasks and into 5 L of acarbose synthesis medium in a 7.5 L fermenter (INFORS). The cultures were grown at 28°C, with shaking at 200 rpm for the 500 mL flasks, and with shaking at 400 rpm for the 7.5 L fermenter.

### Biomass composition

The biomass components of *Actinoplanes* sp. consist of protein, DNA, RNA, lipids, cell wall constituents (peptidoglycan, carbohydrates, and teichoic acid), and small molecules. The biomass equation is important to obtain a high-quality model (Rocha et al., 2008), and because there is no detailed information about the biomass composition, it was partly measured and partly referenced in the literature. The cells were collected during the mid-exponential growth phase, washed three times with distilled water, and lyophilized. To determine the amino acid composition, cell pellets with a dry weight of 250 mg were hydrolyzed using HCl, and the hydrolysates were analyzed by HPLC (Christias et al., 1975). The Coomassie brilliant blue method was used to measure the protein content. Total DNA was determined by the method of Burton (1955). Total RNA was determined by the method of Benthin (Benthid et al., 1991). Lipids were extracted and measured as described as Izard (Izard and Limberger, 2003). The DNA composition was calculated by using the G+C content (71.3%) (Schwientek et al., 2012). The RNA composition was calculated based on the fact that the presumptive percent of mRNA, rRNA, and tRNA was 5, 75, and 20%, respectively, and the nucleotide sequences for rRNAs and tRNAs were derived from published genome information (Schwientek et al., 2012). The phospholipid composition was adopted from *Mycobacterium tuberculosis* (Nandedkar, 1983), and the fatty acid composition was adopted from *Actinoplanes* species (Ara et al., 2010). The precursors demand for triacylglycerols, peptidoglycan, and teichoic acid were assumed to be same as those of *Streptomyces coelicolor* A3(2) (Borodina et al., 2005).

### Analytical methods

The dry cell weight (DCW) was measured by filtration: 15 mL of culture was filtered via a vacuum pump, followed by washing three times with distilled water and drying at 70°C overnight to a constant weight. The acarbose concentration was measured by HPLC using a Thermo Fisher Scientific (Waltham, MA, USA) Hypersil APS-2 NH_2_ column (250 × 4.6 mm, 5 μm). The column temperature was maintained at 40°C, the UV detector was set at 210 nm, and the mobile phase was composed of acetonitrile and phosphate buffer (0.3 g/L KH_2_PO_4_ and 0.35 g/L Na_2_HPO_4_) at a volumetric ratio of 70:30, and run at 1.0 mL/min. The residual maltose concentration was determined by the 3,5-dinitrosalicylic acid method. The concentration of ammonium was determined based on the Berthelot reaction.

### Metabolic network simulation and analysis

The resulting model was analyzed using COBRA Toolbox-2.0 (Schellenberger et al., 2011) through the MATLAB interface. For growth simulation, the biomass equation in a minimal medium was set as the objective function. The essential gene analysis was conducted using the “singleGeneDeletion” function of the COBRA toolbox; if the grRatio was lower than 10^−6^, then the gene was defined as an essential gene. For simulation of acarbose production, the biomass was set to a fixed value, and the acarbose exchange reaction was set as the objective function. The identification of gene amplification targets was based on the strategy of flux scanning based on enforced objective flux (FSEOF) (Choi et al., 2010). The effect of dissolved oxygen (DO) was analyzed by robustness analysis with the fitted substrate uptake rate and cell growth rate (0.025 h^−1^) as the constraints to predict the acarbose production rate at each controlled oxygen uptake rate. Each amino acid was constrained to have a maximum consumption rate of 0.001 mmol/g DCW/h when simulating the effects of amino acids on acarbose production and cell growth, and the biomass equation and acarbose exchange reaction were set as the objective function, respectively.

## Results

### Reconstruction and properties of model *i*YLW1028

**Figure 1:**
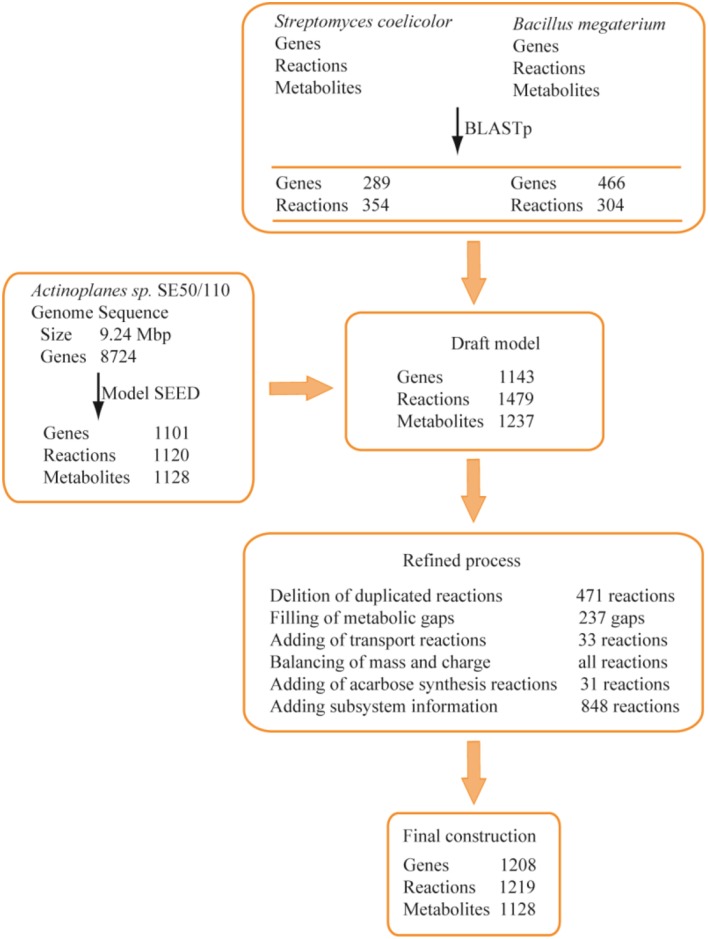
**Procedure of metabolic model reconstruction**. In BLASTp, genes mean the matched genes between the two genomes. In Model SEED, genes and reactions were obtained from auto-reconstructed procedure.

The reconstruction of the GSMM was completed with an automated procedure and manual refinement. As described in Figure 1, an auto-reconstructed model was generated from the Model-SEED database, and a reaction list was obtained through BLASTp. After the integration of the auto-reconstructed model and BLASTp reaction lists, a draft model, including 1479 reactions and 1143 genes, was obtained. The draft model was further refined by comparison with public databases and the literature (Brunkhorst and Schneider, 2005; Licht et al., 2011; Wendler et al., 2013), including deleting duplicated reactions, filling gaps, adding transport reactions, balancing of mass and charge, adding acarbose synthesis reactions, and adding subsystem information. Thirty one synthesis reactions of acarbose were added to the draft model by literature mining (references were shown in Supplementary Data Sheet 2). Thirty three transport reactions were added according to the KEGG (http://www.genome.jp/kegg/), TCDB (http://www.tcdb.org/), and extensive literature mining to obtain a better understanding of substrate utilization. For example, various sugar transport reactions and metabolic pathways were checked and added to the draft model. The maltose/maltotriose ABC transporters *aglEFG*, which were not included in draft model, were added by literature mining (Brunkhorst and Schneider, 2005; Wendler et al., 2013). In addition, the transport reactions of dextrin, galactose, sucrose, and trehalose were added to the draft model according to literature mining (Brunkhorst and Schneider, 2005) and TCDB.

**Figure 2:**
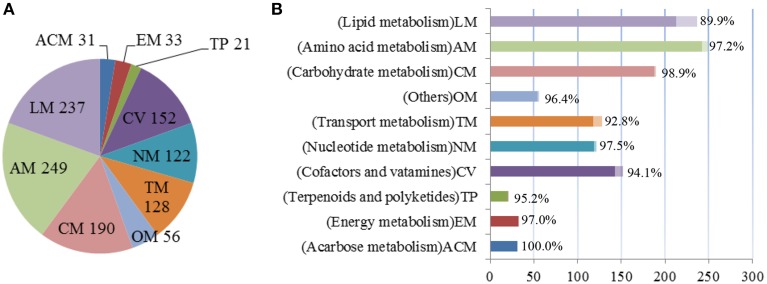
**Properties of model** ***i*****YLW1028. (A)** The numbers of reactions in each subsystem. **(B)** The percent of reactions associated with genes in each subsystem.

The final GSMM, *i*YLW1028, consists of 1219 reactions, 1028 genes, and 1128 metabolites (Data Sheet 1 in Supplementary Materials). As shown in Table 2, 12.5% of the total open reading frames (ORFs), corresponding to 1028 genes of 8247 ORFs, were incorporated into the model. The 1219 reactions were classified into 10 different subsystems (65 metabolic pathways) and located into two cellular compartments (intracellular and extracellular) according to the CELLO database. The numbers of reactions in each subsystem can be seen in Figure 2A. Amino acid metabolism was the largest subsystem, followed by lipid metabolism and carbohydrate metabolism. The sum of these three largest subsystems accounted for more than 50% of the total number of reactions. There were 1162 gene-associated reactions in all, and more than 90% of the subsystems, except for lipid metabolism, were associated with genes, as shown in Figure 2B.

**Table 2:** **Comparison of genome characteristics and model characteristics**.

**Model parameters**	***Actinoplanes* sp. SE50/110 (*i*YLW1028)**	***S. coelicolor* A3(2) Borodina et al., 2005**	***M. tuberculosis* H37Rv Jamshidi and Palsson, 2007**
**GENOME FEATURE**			
Genome size (Mbp)	9.24	8.7	4.41
Total open reading frames	8247	7825	3924
G+C content (%)	71.36	72.12	65.6
**GENOME-SCALE METABOLIC MODEL FEATURE**			
Total reactions	1219	699	936
Biochemical transformations	1091	560	843
Transport reactions	128	139	93
Metabolites	1128	500	740
ORFs assigned in metabolic network	1028	711	661
ORF coverage (%)	12.5	9.1	16.8
Compartments	2 (c, e)	2 (c, e)	2 (c, e)

To understand the characteristics of model *i*YLW1028, a comparison of model *i*YLW1028 with models of *Streptomyces coelicolor* A3(2) and *Mycobacterium tuberculosis* H37Rv, which all belong to the *Actinomycetales*, was also conducted (Table 2). The coverage of the annotated ORFs in the three models (*Actinoplanes* sp. SE50/110, *S. coelicolor* A3(2), and *M. tuberculosis* H37Rv) was 12.5, 9.1, and 16.8%, respectively. Additionally, 265 reactions were shared by the three models. The sum of reactions in carbohydrate metabolism, amino acid metabolism, the metabolism of cofactors and vitamins, and nucleotide metabolism accounted for more than 85% of the 265 shared reactions. There were 330 metabolites shared by the three models, and the number of unique metabolites in the three models (*Actinoplanes* sp. SE50/110, *S. coelicolor* A3(2), and *M. tuberculosis* H37Rv) were 502, 94, and 95, respectively. Model *i*YLW1028 had more abundant unique metabolites than the other two models, demonstrating that it was more comprehensive.

### Determination of biomass composition and verification of model *i*YLW1028

Because no detailed biomass information was available for *Actinoplanes* sp. SE50/110, the biomass composition was determined and referenced from the literature of related species. For biomass composition measuring, strain *Actinoplanes* sp. SE50/110 was collected during the mid-exponential growth phase, washed three times with distilled water, and lyophilized and measured as detailed in the Methods section. The total biomass was composed of 58% protein, 1% DNA, 11% RNA, 5% lipids, 22% cell wall, and 3% small molecules. The biomass equation and their compositions are shown in Supplementary Data Sheet 2. For all simulations in model *i*YLW1028, the biomass compositions were assumed to be independent of the environmental conditions.

The capability of utilizing 40 different carbon sources (12 saccharides, 4 alcohols, 4 carboxylic acids, 20 amino acids) for cell growth was qualitatively predicted by flux balance analysis. Each carbon source was used as the sole carbon source in a minimal medium, and the results were compared to experimental data, as shown in Table 3. A 92% match was obtained. This indicated there were no fatal gaps in model *i*YLW1028 and that model *i*YLW1028 could predict the catabolic pathways of various carbon sources. Three inconsistent cases were inositol, mannitol, and acarbose. The reasons for the three inconsistent results are: (1) genes in the metabolic pathways of inositol and mannitol were not annotated, so the metabolic pathways were interrupted; (2) *Actinoplanes* sp. SE50/110 could grow on acarbose minimal medium, and the reason for acarbose was that its transport into the cytoplasm was unclear (Brunkhorst and Schneider, 2005). In addition, of the 40 different carbon sources, 37 carbon sources could sustain cell growth, which indicates that *Actinoplanes* sp. SE50/110 has a wide substrate utilization spectrum.

**Table 3:** **Growth phenotypic validation under a sole carbon source**.

**Substrate**		**Biomass**		
**Carbon source**		***In vivo***	***In silico***	**References**
SACCHARIDE	Glucose	+	+	This study
	Sucrose	+	+	This study
	Maltose	+	+	This study
	Dextrin	+	+	This study
	Starch	+	+	This study
	Fructose	+	+	This study
	Lactose	+	+	Frommer et al., 1971; Brunkhorst and Schneider, 2005
	Xylose	+	+	This study
	Trehalose	+	+	This study
	Galactose	+	+	Frommer et al., 1971
	Acarbose	−	+	Brunkhorst and Schneider, 2005
	Arabinose	+	+	This study
ALCOHOL	Glycerol	+	+	This study
	Mannitol	+	−	This study
	Sorbitol	+	+	Frommer et al., 1971
	Inositol	+	−	Frommer et al., 1971
CARBOXYLIC ACID	Pyruvate	+	+	This study
	Acetate	+	+	This study
	Citrate	+	+	This study
	Malic acid	+	+	This study
AMINO ACIDS	L−Alanine	+	+	This study
	L-Glutamate	+	+	This study
	L-Glutamine	+	+	This study
	L-Glycine	+	+	This study
	L-Threonine	+	+	This study
	L-Aspartate	+	+	This study
	L-Asparagine	−	−	This study
	L-Tryptophan	−	−	This study
	L-Histidine	+	+	This study
	L-Serine	+	+	This study
	L-Tyrosine	−	−	This study
	L-Valine	+	+	This study
	L-Lysine	−	−	This study
	L-Arginine	+	+	This study
	L-Cysteine	−	−	This study
	L-Proline	+	+	This study
	L-Phenylalanine	−	−	This study
	L-Leucine	+	+	This study
	L-Isoleucine	+	+	This study
	L-Methionine	−	−	This study

The capability of utilizing different nitrogen sources for cell growth was also predicted by flux balance analysis. It contained 23 different nitrogen sources (ammonium, nitrate, urea, and 20 amino acids). The results were compared to experimental data, as shown in Table 4. The main results are as follows. (1) A match of 91% between *in vivo* and *in silico* was obtained. Of the 23 nitrogen sources, *Actinoplanes* sp. SE50/110 could grow on 21 sources. Two inconsistent cases were tryptophan and phenylalanine. (2) Phenylalanine could not sustain cell growth in model *i*YLW1028 because genes in the phenylalanine metabolic pathway were not annotated. (3) Tryptophan as the sole nitrogen source could not sustain growth because the genes of the intermediate metabolite (2-hydroxyphenylacetate) metabolic pathway were not annotated.

**Table 4:** **Growth phenotypic validation under a sole nitrogen source**.

**Substrate**	**Biomass**		
**Nitrogen source**	***In vivo***	***In silico***	**References**
(NH_4_)_2_SO_4_	+	+	This study
NaNO_3_	+	+	This study
Urea	+	+	This study
L-Alanine	+	+	This study
L-Glutamate	+	+	This study
L-Glutamine	+	+	This study
L-Glycine	+	+	This study
L-Threonine	+	+	This study
L-Aspartate	+	+	This study
L-Asparagine	−	−	This study
L-Tryptophan	+	−	This study
L-Histidine	+	+	This study
L-Serine	+	+	This study
L-Tyrosine	−	−	This study
L-Valine	+	+	This study
L-Lysine	−	−	This study
L-Arginine	+	+	This study
L-Cysteine	−	−	This study
L-Proline	+	+	This study
L- Phenylalanine	+	−	This study
L-Leucine	+	+	This study
L-Isoleucine	+	+	This study
L-Methionine	−	−	This study

Batch fermentation with different maltose uptake rates (fitted from different original maltose concentration data) using acarbose synthesis medium was conducted to evaluate model *i*YLW1028 quantitatively. The fermentation data were used as constraints to simulate cell growth, including the specific growth rate (μ), maltose uptake rate (MUR), ammonium uptake rate (AUR), and acarbose production rate (APR). As shown in Table 5, the growth rate *in silico* agreed with the fermentation data. In addition, cell growth on 9 carbon source (glucose, sucrose, galactose, fructose, trehalose, lactose, arabinose, glycerol, and sorbitol) and 2 nitrogen source (urea and glutamate) under the minimal medium was also used for quantitative validation of model *i*YLW1028. The *in silico* growth rates were very close to the *in vivo* values, and they were lower than 10% of the experimental growth rates. The *in silico* and *in vivo* specific growth rates and all the cellular flux on the 9 carbon sources and 2 nitrogen sources were listed in Supplementary Data Sheet 5. The agreement of *in silico* and *in vivo* growth rates indicates that model *i*YLW1028 could successfully reflect the cellular metabolism of *Actinoplanes* sp. SE50/110.

**Table 5:** **Comparison between the simulation value and fermentation data with different experimental constraints by *Actinoplanes* sp. SE50/110**.

**Constraints (mmol/gDCW/h)**			**Growth rate (h**^−^**^1^)**	
**MUR**	**AUR**	**APR**	***In vivo***	***In silico***
0.17	0.23	0.0024	0.026	0.024
0.26	0.31	0.0054	0.034	0.032
0.27	0.35	0.0035	0.038	0.037

### Essential genes and reactions for cell growth

**Figure 3:**
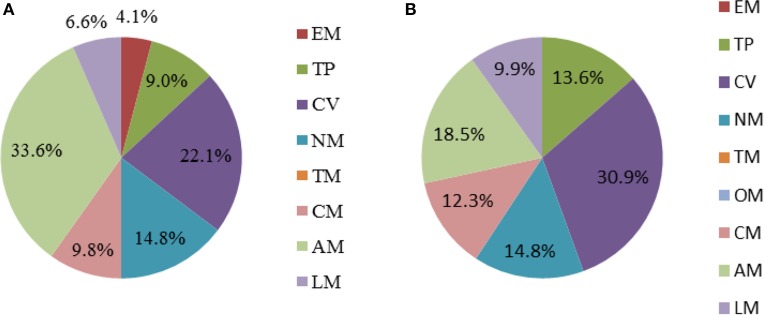
**Percentage of essential genes in each subsystem under different cultivated conditions. (A)** Acarbose-synthesis medium. **(B)** Sucrose medium. The abbreviations of each subsystem are the same with that in Figure 2.

The analysis of genes essential for cell growth was performed by single-gene deletion through MATLAB in two media (acarbose synthesis medium and sucrose medium). As described in Figures 3A,B, 122 genes (11.9%) were predicted to be essential in acarbose synthesis medium, and 81 genes (7.9%) were predicted to be essential in sucrose medium (all the essential genes and simulated conditions are listed in Supplementary Data Sheet 3). More than 75% of the essential genes were concentrated in four subsystems (amino acid metabolism, the metabolism of cofactors and vitamins, nucleotide metabolism, carbohydrate metabolism) under the two conditions, demonstrating that these subsystems play a significant role in cell growth. Additionally, all genes essential for growth in sucrose medium were included in those needed for growth in acarbose synthesis medium, although there was a considerable difference between the two media. The functions of unique genes in acarbose synthesis medium are shown in Supplementary Data Sheet 3. Most of the unique genes participated in amino acid synthesis directly or by supplying precursors. For example, gene ACPL_8142 participated in L-tyrosine synthesis, and genes ACPL_8304 and ACPL_887 participated in L-phenylalanine synthesis. In addition, genes ACPL_1328 and ACPL_1861 belong to the pentose phosphate pathway, but there was no flux of the pentose phosphate pathway in sucrose medium. When a BLASTp search was performed with the database of essential genes (Luo et al., 2014), a 90% match was acquired, which indicated the accuracy of model *i*YLW1028. However, 2 of 11 unmatched genes were found to be essential in *S. coelicolor* A3(2). A protein encoded by gene ACPL_1709 and SCO2103 (an essential gene in *S. coelicolor* A3(2)) catalyzed the formation of 5-methyltetrahydrofolate from 5,10-methylenetetrahydrofolate. Proteins encoded by genes ACPL_6471 and SCO1481 (an essential gene in *S. coelicolor* A3(2)) also had the same function, as they catalyzed the transfer of orotidylic acid to uridine monophosphate (UMP).

**Figure 4:**
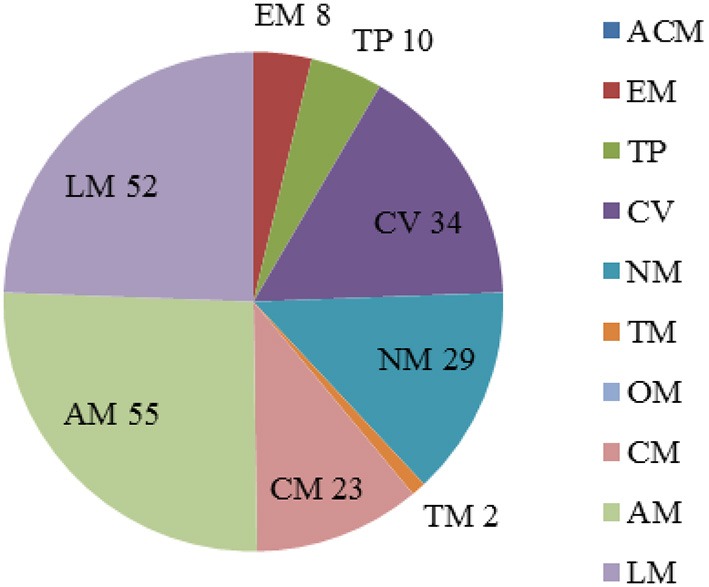
**Percentage of essential reactions in each subsystem with maltose as the sole carbon course**. The abbreviations of each subsystem are the same with that in Figure 2.

The analysis of essential reactions was performed with the same method in acarbose synthesis medium. There were 213 reactions that were predicted to be essential for cell growth (Data Sheet 3 in Supplementary Materials). The pathways of the essential reactions can be seen in Figure 4, and almost 80% of the essential reactions were found in pathways involved in the metabolism of cofactors and vitamins (34 reactions), nucleotide metabolism (29 reactions), amino acid metabolism (55 reactions), and lipid metabolism (52 reactions). Of the 114 genes in essential reactions, 113 belong to essential genes that were described previously.

### The acarbose biosynthetic pathway in *Actinoplanes* sp. SE50/110

**Figure 5:**
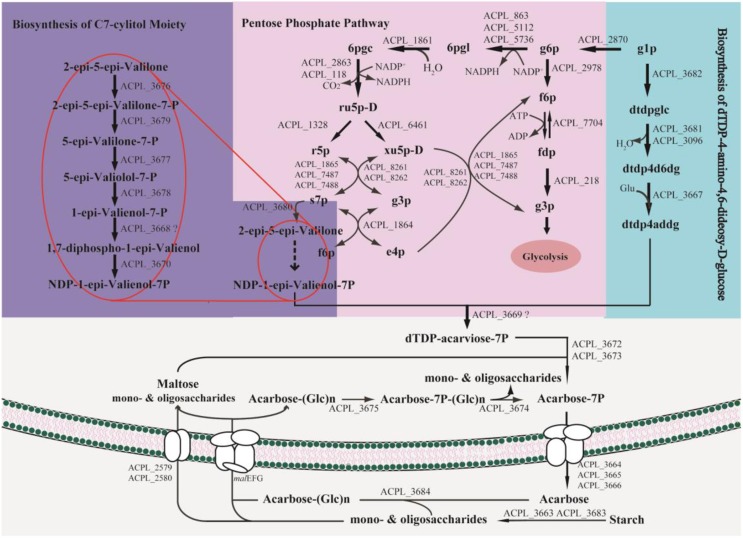
**Biosynthetic pathways of acarbose in *Actinoplanes* sp. SE50/110**. The genes *mal*EFG in the pathway were ACPL_6402, ACPL_3200, ACPL_4618, ACPL_5585, ACPL_6401, ACPL_6726, ACPL_3782, ACPL_5386, and ACPL_6400.

**Figure 6:**
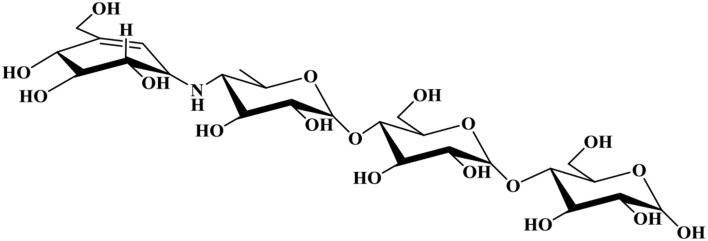
**Chemical structure for acarbose**.

Model *i*YLW1028 consists of 31 reactions of acarbose biosynthesis (Figure 5) and the chemical structure for acarbose was as shown in Figure 6. The acarbose biosynthetic pathway was classified into three fractions. (1) Biosynthesis of C7-cylitol moiety: first, 2-epi-5-epi-valiolone synthase, encoded by ACPL_3680, catalyzed the transfer of sedoheptulose 7-phosphate to 2-epi-5-epi-valiolone. Then, after other six enzymatic reactions encoded by ACPL_3668, ACPL_3670, and ACPL_3676–3679, 2-epi-5-epi-valiolone was converted to NDP-1-epi-valienol 7-phpsphate. (2) Biosynthesis of dTDP-4-amino-4,6-dideoxy-D-glucose: it begins with glucose 1-phsophate. There are three reactions in the biosynthesis pathway of dTDP-4-amino-4,6-dideoxy-D-glucose, in which enzyme AcbV is an L-glutamic acid-dependent enzyme. (3) The formation of acarbose and its transport: following catalysis by the deduced protein AcbS encoded by ACPL_3669, NDP-1-epi-valienol 7-phosphate and dTDP-4-amino-4,6-dideoxy-D-glucose are linked to form dTDP-acarviose 7-phosphate, which was further modified by the deduced proteins AcbI and AcbJ (encoded by ACPL_367 and ACPL_3673), leading to the formation of acarbose 7-phosphate. Acarbose 7-phosphate was assumed to be the end-product of the cytoplasmic pathway. With the help of an ABC transporter encoded by ACPL_3664–3666, acarbose 7-phosphate was transported out of the cell, leading to the formation of acarbose. In addition, model *i*YLW1028 illustrated a “carbophor,” a possible cycling of acarbose-derived metabolites between intracellular and extracellular pools. The extracellular acarbose could link with extracellular saccharides, such as glucose and maltose, which may hydrolyzed by α-amylase (encoded by ACPL_3663 and ACPL_3683) from starch, dextrin, and so on, and then transported into the cell, followed by phosphorylation by acarbose-7-kinase. The intracellular acarbose-derived metabolites were deglycosylated by an amylomaltase-equivalent enzyme (encoded by ACPL_3674), which could form a cycle. Acarbose inhibits the α-glycosidases of competitors via the carbophor.

The essential genes for acarbose production were studied in acarbose synthesis medium. One hundred and thirty three genes were essential for acarbose synthesis, and compared with the essential genes for cell growth, there were 15 unique genes, which all belong to *acb* cluster genes. All these 15 *acb* cluster genes were essential for acarbose production, and the deletion of any gene prevented the production of acarbose. The 7 *acb* clusters genes that were non-essential for acarbose production in model *i*YLW1028 were ACPL_3663 (*acbZ*), ACPL_3683 (*acbE*), ACPL_3674 (*acbQ*), ACPL_3675 (*acbK*), ACPL_3684 (*acbD*), ACPL_3671 (*acbP*), and ACPL_3681 (*acbB*). All of these genes contribute to acarbose production, except for ACPL_3681, and they are essential genes. The reasons are as described below. (1) Genes ACPL_3675, ACPL_3674, and ACPL_3684 participated in the reaction of the “carbophor.” However, model *i*YLW1028 failed to simulate the function of the “carbophor,” so these three genes were silent in model *i*YLW1028. (2) ACPL_3681 and ACPL_3096 had moderate similarities of 52% (Schwientek et al., 2012) and the proteins encoded by both genes can catalyze the formation of dTDP-4-keto-6-deoxy-D-glucose. Thus, gene ACPL_3681 belongs to the *acb* cluster, but is not essential for acarbose production. (3) The enzymes encoded by ACPL_3683 and ACPL_3663 hydrolyze starch to glucose; thus, in model *i*YLW1028, they were predicted to be non-essential genes for acarbose production without the addition of starch. (4) The function of ACPL_3671 is still unknown.

There are four notable aspects in the acarbose synthesis pathway. (1) The starting precursor of the acarbose biosynthetic pathway is 2-epi-5-epi-valiolone, which is derived from sedoheptulose 7-phosphate. Sedoheptulose 7-phosphate is an intermediate metabolite of the pentose phosphate pathway. (2) Glutamate is the primary source of the nitrogen in acarbose. Glutamate and dTDP-4-keto-6-deoxy-D-glucose formed dTDP-4-amino-4,6-dideoxy-D-glucose following catalysis by AcbV, thus introducing the nitrogen into acarbose. (3) Because the starting precursor of acarbose derived from the pentose phosphate pathway, and NADPH was formed along with the pentose phosphate pathway, the cofactor (NADPH) regulation was predicted to affect acarbose production. (4) The expression of the acarbose cluster genes was different in different cultivation media (Schwientek et al., 2013); thus, the overexpression of the *acb* cluster genes would contribute to acarbose production.

### *In silico* simulation of acarbose production

**Figure 7:**
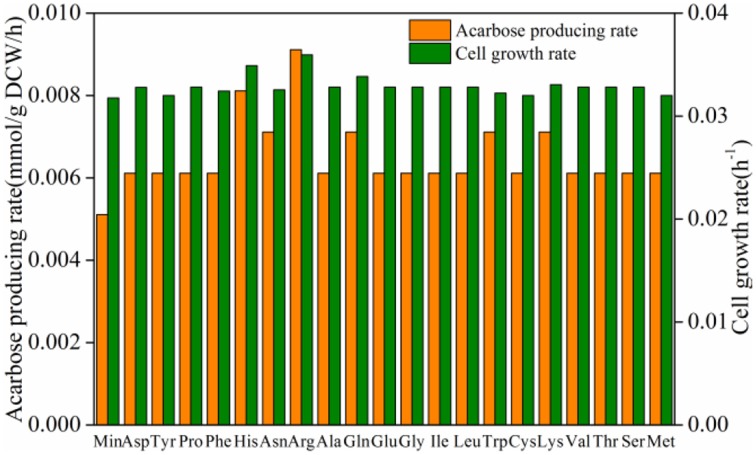
**Effect of amino acids on cell growth rate and acarbose production rate**.

To increase the production of acarbose, three types of efforts were made. In model *i*YLW1028, the effect of additional amino acids on cell growth and acarbose production was simulated (Figure 7). The results show that the addition of amino acids had a good effect on both cell growth and the acarbose production rate. The addition of arginine had the most significantly impact on acarbose production, followed by histidine. And compared to the control, the addition of the aforementioned two amino acids increased the acarbose production rate by 78 and 59%, respectively.

To find candidate genes to be overexpressed to increase acarbose production, the strategy of FSEOF was applied. When the acarbose production rate was increased gradually, four types of flux profiles were identified: increased, decreased, irregular, and unchanged flux. The flux profile is shown in Supplementary Data Sheet 4. The ratio of pentose phosphate pathway flux to glycolysis flux increased with increasing acarbose production. This simulation result reveals that acarbose production increased with the enhancement of the pentose phosphate pathway and the inhibition of glycolysis. Thus, genes in the pentose phosphate pathway, such as ACPL_1861, ACPL_6461, ACPL_1328, were candidates for overexpression to improve acarbose production. In addition, the flux of the reaction catalyzed by alanine aminotransferase (EC: 2.6.1.2), which forms glutamate, increased with increasing acarbose production. Thus, ACPL_6750 was another candidate gene to be overexpressed.

**Figure 8:**
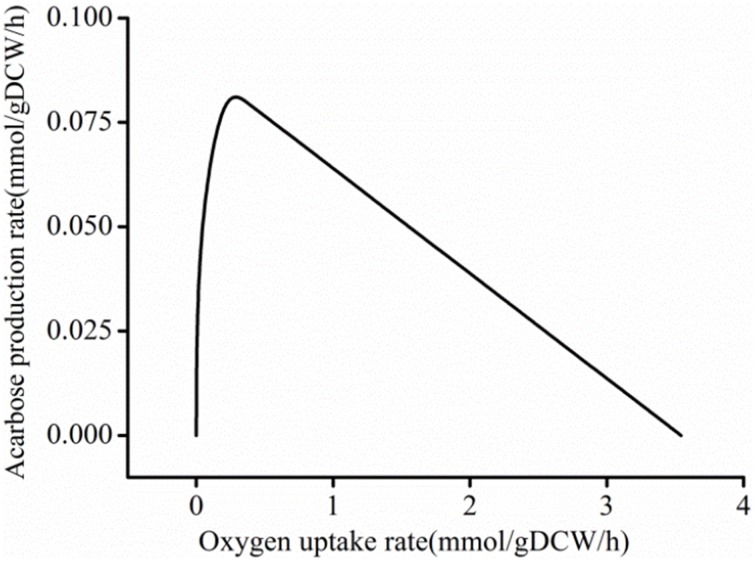
**Robustness analysis of oxygen uptake rate**.

Meanwhile, oxygen flux was reduced during the whole FSEOF strategy. Therefore, to gain insight into the effect of the oxygen uptake rate on acarbose production, the influence of the oxygen uptake rate on acarbose formation was studied by robustness analysis in acarbose synthesis medium. When the maltose and ammonium uptake rate were constrained to be 0.269 and 0.330 mmol/g DCW/h, respectively, and biomass was constrained to 0.025 h^−1^, it was found that the acarbose production rate increased sharply when the oxygen uptake rate was in the range of 0–0.2 mmol/g DCW/h (Figure 8). However, when the oxygen uptake rate exceeded 0.2 mmol/g DCW/h, the acarbose production rate began to decrease, and eventually reached zero as the oxygen uptake rate increased.

During fermentation, a small number of other acarbose-like by-products (component A–H) were also produced, of which component C is the most difficult impurity to separate during downstream processing. Component C was produced from acarbose by TreY (Lee et al., 2008). Because TreY was predicted to be a cytoplasmic enzyme according to CELLO in this work, and acarbose existed in a phosphorylated form, in model *i*YLW1028, component C was supposed to be formed from acarbose 7-phosphate. Gene *treY* was not an essential gene for growth and acarbose synthesis. Thus, the deletion of *treY* could contribute to acarbose production and downstream processing, and because two other trehalose synthesis pathways (Lee et al., 2008) exist, the deletion of *treY* will not have an effect on trehalose synthesis, which is essential for cell growth (Di Lernia et al., 1998; Avonce et al., 2006; Lee et al., 2008). This prediction was also supported by a previous study, in which *treY* was inactivated in *Actinoplanes* sp. 8–22 and component C was decreased by 66.7% (Huang et al., 2013).

### Effects of dissolved oxygen on acarbose production

**Figure 9:**
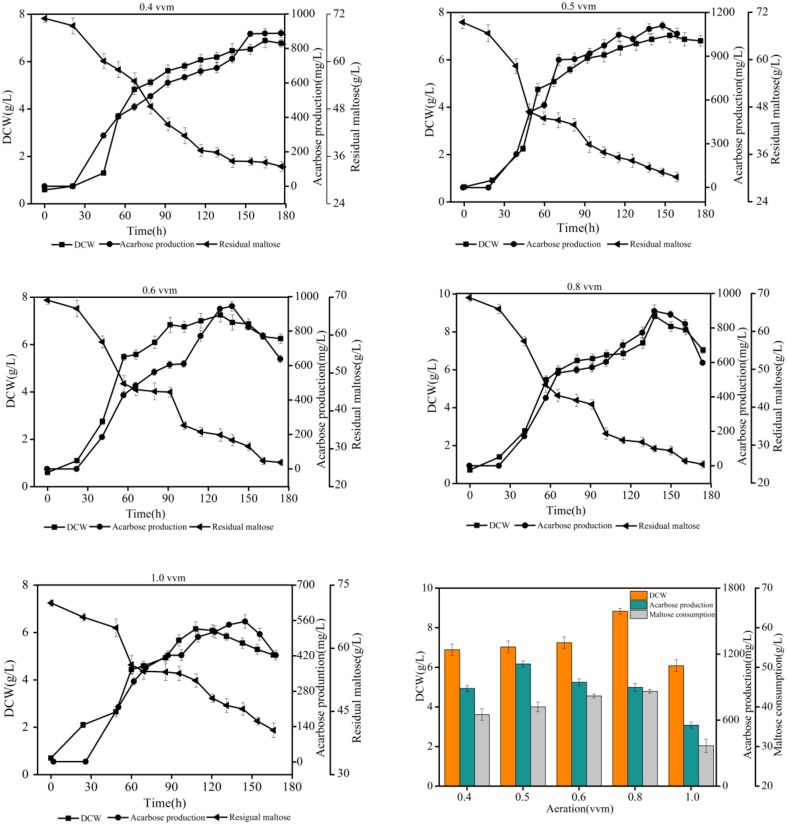
**Effects of different aeration rates on acarbose production**.

To gain insight into the effect of dissolved oxygen (DO) on acarbose production in batch fermentation experiments, a series of experiments under different aeration rates [0.4, 0.5, 0.6, 0.8, and 1.0 volume vs. mass (vvm)] were conducted. The agitation speed was set at 400 rpm during the whole period of fermentation. The results of the batch fermentation are shown in Figure 9. As a result, the final acarbose concentration (mg/L) and yield (g/g DCW) increased by increasing the aeration rate to 0.5 vvm, but further increasing the aeration rate decreased the final acarbose concentration and yield. The highest DCW and maltose consumption were observed at 0.8 vvm. Under this condition, most consumption of the carbon source was used to synthesize biomass, not acarbose. When aeration was further increased to 1.0 vvm, the DCW, acarbose production, and maltose consumption decreased. These results are a strong indication that excessive oxygen exposure during fermentation inhibits acarbose accumulation, while a relatively low DO level is good for acarbose production.

## Discussion

*Actinoplanes* sp. SE50/110 is known to be the wild-type producer of the α-glucosidase inhibitor acarbose (Frommer et al., 1977). This study presents the first GSMM for *Actinoplanes*. Model *i*YLW1028 was reconstructed based on iterative measures involving literature mining, the genome sequence, and manual refinement. The capability of utilizing different carbon/nitrogen sources and the agreement between *in silico* and *in vivo* cell growth rates on different carbon/nitrogen sources indicated the accuracy of model *i*YLW1028. Based on model *i*YLW1028, strategies to improve acarbose production were simulated. It was indicated that, additional amino acids had a beneficial effect on acarbose production, especially L-arginine and L-histidine, and that a relatively low DO level was also beneficial for acarbose production. Gene targets who overexpression could increase the levels of acarbose precursors were identified via the FSEOF algorithm. In addition, the deletion of *treY* could eliminate the producing of by-product component C. Based on model *i*YLW1028 and simulation, it is possible to predict limiting factors in the acarbose biosynthetic pathway, perform metabolic engineering to improve acarbose production, and interpret the mechanism at the systems level.

In previous studies, many efforts have been made to interpret the acarbose biosynthetic pathway (Lee et al., 1997; Lee and Egelkrout, 1998; Mahmud et al., 1999; Zhang et al., 2002) and the function of *acb* clusters genes (Stratmann et al., 1999; Hemker et al., 2001; Zhang et al., 2003; Licht et al., 2011). In model *i*YLW1028, the reactions in acarbose biosynthetic pathway were summarized and illustrated at a system level to systematically aid our understanding of the biosynthetic pathway. It started with sedoheptulose 7-phosphate and was classified into three fractions: biosynthesis of the C7-cylitol moiety, biosynthesis of dTDP-4-amino-4,6-dideoxy-D-glucose, and the formation of acarbose and its transport. To increase acarbose production, the key factors limiting acarbose production have been discovered and a series of efforts have been made. Because maltose is directly incorporated into acarbose (Lee et al., 1997), the type of carbon source in the fermentation medium is a limiting factor, and it has been optimized. The carbon sources and concentrations that promoted acarbose production in *Actinoplanes* sp. A56 are 30 g/L glucose and 61.25 g/L maltose (Wei et al., 2010). Osmolality was another limiting factor (Beunink et al., 1997; Wang et al., 2012). Maintaining an elevated osmolality via intermittent feeding of necessary components (14.0 g/L maltose, 6.0 g/L glucose, and 9.0 g/L soybean meal, with feeding at 48, 72, 96, and 120 h and a feed volume of 5 mL) during the fermentation of *Actinoplanes utahensis* ZJB-08196 increased acarbose production by 15.9%. In this study, two limiting factors were identified based on model *i*YLW1028. First, the flux of the ratio of the carbon source flow to pentose phosphate pathway was 13.2% according to the analysis of the distribution of carbon sources. Because the direct precursor of acarbose comes from the pentose phosphate pathway, the low level of this intracellular precursor was considered to be a limiting factor. Second, through the robustness analysis of the oxygen uptake rate, it was shown that the acarbose producing rate was affected by the oxygen uptake rate, which was another limiting factor.

Recent strategies to improve acarbose production focused on fermentation compositions (Choi and Shin, 2003; Wang et al., 2011; Sun et al., 2012) and culture conditions (Wang et al., 2011; Li et al., 2012; Cheng et al., 2014), and they have increased acarbose production considerably. However, very little is known about the overexpression or knockout of gene targets to improve acarbose production. Based on the GSMM in this study, we simulated gene overexpression and gene knockouts to guide metabolic engineering to improve acarbose production. It was shown that the overexpression of genes ACPL_1861 (6-phosphoglucanolactonase), ACPL_6461 (ribulose phosphate 3-epimerase), ACPL_1328 (ribose 5-phosphate isomerase) and ACPL_6750 (aminotransferase) promoted acarbose producing. However, no candidate genes to be knockout were found through single and double gene deletions. The overexpression of these four genes lead to a high level of intracellular precursors, which is in agreement with the aforementioned limiting factor. Genes ACPL_1861, ACPL_6461, and ACPL_1328 participated in the pentose phosphate pathway, the overexpression of which increased the precursors of the pentose phosphate pathway. The expression of gene ACPL_6750 would increase the formation of glutamate. Although the wet lab experiments were not completed in our study, the effect of genes ACPL_1861 and ACPL_6461 on the metabolic pathway were supported by literature mining. (1) The deletion of gene *devB* (6-phosphoglucanolactonase) (Butler et al., 2002) in *Streptomyces lividans* resulted in a significantly higher antibiotic productivity and a reduced level of pentose phosphate pathway flux. This indicates that overexpression of gene ACPL_1861 might show an increased flux of the pentose phosphate pathway, which could have a beneficial effect on acarbose production. In addition, by overexpressing of gene *pgl* (encoding 6-phosphogluconolactonase), the riboflavin titer of *E. coli* RF01S/p15Trc-zgp (Lin et al., 2014) increased by 15.7% compared with strain RF01S/p15Trc-zg, which indicates an enhanced flux of the pentose phosphate pathway. (2) The deletion of gene *rpe* (ribulose phosphate 3-epimerase) (Shimaoka et al., 2005) in *E. coli* resulted in low cell growth. It is known that the overall demand for NADPH for biosynthesis is >80% of total cytosolic NADPH production (Fan et al., 2014), and that the most direct route to produce NADPH is via the pentose phosphate pathway. Thus the deletion of *rpe* might reduce the flux through the pentose phosphate pathway. However, due to the imperfect genetic manipulation of *Actinoplanes* sp. SE50/110, including the lack of suitable expression vector and transformation method with a high frequency, the wet experiments of gene overexpression are not conducted, which is a weakness of verification on model *i*YLW1028. In addition, since the effect of the four simulated genes on acarbose production was not verified by wet experiment, they are not appropriate to apply to acarbose producing strains at the present stage. Therefore, Future work for improving acarbose production should focus on the overexpression of these four genes.

The result of the robustness analysis indicated that a relatively lower oxygen uptake rate promoted acarbose production. Additionally, it is known that the DO level plays an important role in acarbose fermentation by *Actinoplanes* sp. (Li et al., 2012). To elucidate the mechanism by which the oxygen uptake rate affects the acarbose production rate, the distributions of carbon flux under different oxygen levels (aerobic and semi-aerobic) were investigated. It was found that the ratio of pentose phosphate pathway flux to glycolytic flux to was obviously affected by the oxygen levels, as the ratio increased by about 27% when the cells were shifted from a semi-aerobic to aerobic environment. The simulated result was proved by wet lab experiments using different aeration rates. In addition, it has been shown that AcbV, which is required for the biosynthesis of dTDP-4-amino-4,6-dideoxy-D-glucose is an L-glutamic acid-dependent enzyme (Piepersberg et al., 2002; Wehmeier, 2003). Furthermore, in resting cell experiments, several experimental amino acids were found to be a preferred nitrogen source (Lee and Egelkrout, 1998). Therefore the effect of amino acids have been simulated, and it was found that amino acid had a beneficial effect on acarbose production.

In this work, the first GSMM for *Actinoplanes* was successfully used to illustrate the factors thar limit acarbose biosynthetic during fermentation, to simulate gene targets for improved acarbose production, to elucidate the mechanism of the effect of the oxygen uptake rate on acarbose production, and to interpret the effects of amino acids on acarbose. Despite guiding metabolic engineering and fermentation optimization applications, model *i*YLW1028 has many future perspectives. First, while there are large amounts of batch fermentation data, little is known about its dynamic fermentation data. Based on dynamic fermentation data, the predictions of microbial behaviors in a dynamic model would enable the comprehensive understanding of acarbose fermentation. Second, one can integrate high-throughput data with the GSMM. Gene expression data in three different growth media were analyzed via RNA-sequencing (Schwientek et al., 2013), which affected acarbose production greatly. With the gene expression data as constraints, the predictions of model *i*YLW1028 would be more accurate, which would enable us to interpret the different mechanisms of acarbose production in different growth media.

## Conclusion

This study presents the first GSMM for *Actinoplanes*. Model *i*YLW1028 consists of 1219 reactions, 1128 metabolites, and 1028 genes based on genome information, databases, and literature mining. The prediction of metabolism phenotypes and essential genes based on model *i*YLW1028 agreed well with the experimental data and the database of essential genes. Using model *i*YLW1028, *in silico* metabolic fluxes were analyzed to investigate the effect of additional amino acids and DO on acarbose production. The results showed that additional amino acids had a beneficial effect on acarbose production, especially L-arginine and L-histidine, and that a relatively low DO level was also beneficial for acarbose production. The FSEOF algorithm was used to identify targets whose overexpression could increase the level of acarbose precursors, thereby increasing acarbose production directly. We also examined the effect of the deletion of *treY* on growth and trehalose synthesis to eliminate the by-product component C. Hence, it is expected that model *i*YLW1028 will help us understand cellular physiology on a systems level, as well as to perform systems metabolic engineering.

### Conflict of interest statement

The authors declare that the research was conducted in the absence of any commercial or financial relationships that could be construed as a potential conflict of interest.
